# The Impact of Social Isolation, Loneliness, and Technology Use During the COVID-19 Pandemic on Health-Related Quality of Life: Observational Cross-sectional Study

**DOI:** 10.2196/41536

**Published:** 2022-10-19

**Authors:** Eric Balki, Niall Hayes, Carol Holland

**Affiliations:** 1 Division of Health Research Lancaster University Lancaster United Kingdom; 2 Directorate Nottingham Trent University Nottingham United Kingdom

**Keywords:** health-related quality of life, healthy aging, older adult, elder, older person, older population, geriatric, gerontology, technology intervention, COVID-19, pandemic, loneliness, social isolation, isolation, isolated, lonely, cross-sectional, technology use, digital literacy, acceptance

## Introduction

Health-related quality of life (HRQoL), defined as a person’s self-perceived health status in relation to their social, cultural, and environmental context, is linked to better health and the ability to deal with adverse life events [1]. Social factors such as loneliness are known to influence HRQoL negatively [2]. The COVID-19 pandemic has disproportionately impacted older adults, with social distancing measures worsening isolation levels [3], which we hypothesize has resulted in lower levels of HRQoL (hypothesis 1).

Further, technology use is linked to improved self-rated health and psychological well-being, alleviating loneliness among older adults, and encouraging behaviors that may lead to better levels of HRQoL [4]. Digital communication tools became critical during the pandemic to remain socially connected and helped prevent social health risks [5], potentially benefiting those with lower HRQoL [6]. We hypothesized that technology use could predict higher HRQoL (hypothesis 2). Moreover, disease containment measures resulted in increased isolation and loneliness among older adults [3], which could impact HRQoL (hypothesis 3). Increased knowledge about how HRQoL was impacted by pandemic loneliness, isolation, and technology use may better inform health care workers, policy makers, and the public.

## Methods

This was an observational cross-sectional study from March 16, 2020, to June 21, 2021, when social distancing mandates were in force. Participants were recruited in England.

### Ethics Approval

The study received ethical approval from the University Research Ethics Committee (Ref FHMREC19121).

### Participants

Eligible participants were living in their own homes, proficient in English, and aged ≥65 years. The sample (G*Power confirmed effect size of 87) consisted of 89 people aged 65 to 92 (mean 73.2, SD 7.46) years.

### Variables and Measures

Participants completed a background questionnaire capturing age, gender, and ethnicity. We used the following standardized measures: UCLA Loneliness Scale [7], Technology Experience Questionnaire [8], Lubben’s Social Isolation Scale, and Short-Form 36 [9], a measure of HRQoL comprising eight health scales (physical/mental).

### Procedure

Surveys were conducted via telephone, with further analysis done using SPSS Ver 28 (IBM Corp).

### Statistical Methods

Higher scores on the UCLA Loneliness Scale and technology use measures indicated greater loneliness and technology use; lower scores on Lubben’s scale indicated greater isolation. Pearson correlation determined whether lower social isolation (hypothesis 1) and greater technology use (hypothesis 2) were associated with higher HRQoL. Multiple linear regression models were built to evaluate whether loneliness predicted HRQoL after controlling for social isolation and technology use (hypothesis 3).

## Results

Low social isolation (hypothesis 1) and higher technology use (hypothesis 2) were significantly associated with higher HRQoL (Table 1).

Multiple linear regression was calculated (Table 2) for hypothesis 3. Model 1, incorporating loneliness, explained 24.9% of the variance in HRQoL. Model 2, incorporating social isolation, explained an additional nonsignificant 0.1% of the variance (*F*_1,89_=0.112; *P*=.74). Model 3, adding technology use, explained an additional 5.5% of the variance (*F*_1,88_=6.93; *P*=.01).

Semipartial correlations squared showed unique amount of variance; only technology use predicted a significant unique amount of the variance in HRQoL (sr^2^=0.0547; *P*=.01), followed by loneliness (sr^2^=0.0179; *P*=.14) and social isolation (sr^2^=0.0004; *P*=.82).

**Table 1:** Correlational analysis between variables (N=89).

		UCLA Loneliness score	HRQoL^a^	Technology use	Social isolation
**UCLA Loneliness score**					
	Pearson correlation	—^b^	—0.499	–0.631	–0.853
	*P* value	—	<.001	<.001	<.001
**HRQoL**					
	Pearson correlation	−0.499	—	0.497	0.442
	*P* value	<.001	—	<.001	<.001
**Technology use**					
	Pearson correlation	−0.631	0.497	—	0.577
	*P* value	<.001	<.001	—	<.001
**Social isolation**					
	Pearson correlation	−0.853	0.442	0.557	—
	*P* value	<.001	<.001	<.001	—

^a^HRQoL: health-related quality of life.

^b^Not applicable.

**Table 2:** Model output and coefficients of multiple linear regression models for health-related quality of life (N=89).

Independent variables	Model 1			Model 2			Model 3		
	b (SE)	B	*P* value	b (SE)	B	*P* value	b (SE)	B	*P* value
Loneliness	−4.07 (0.745)	−0.499	<.001	−3.66 (1.436)	−0.449	.01	−2.246 (1.490)	−0.275	.14
Social isolation	N/A^a^	N/A	N/A	0.559 (1.671)	0.059	.74	0.369 (1.62)	0.039	.82
Technology use	N/A	N/A	N/A	N/A	N/A	N/A	1.071 (0.407)	0.302	.01
Intercept	757.851 (37.75)	N/A	<.001	723.318 (109.926)	N/A	<.001	536.117 (128.009)	N/A	<.001
*R*^2^ (Δ*R*^2^)	0.249	N/A	<.001	0.250 (0.001)	N/A	.74	0.305 (0.055)	N/A	.01
*F* test (*df*)	29.871 (1,90)	N/A	<.001	14.844 (2,89)	N/A	<.001	12.865 (3,88)	N/A	<.001

^a^N/A: not applicable.

## Discussion

Few studies to date have examined the impact of social isolation, loneliness, and technology use together on HRQoL in older adults in England during the pandemic. We found that loneliness negatively impacts HRQoL, and technology use positively impacts it. Although social isolation has been linked to HRQoL, it had a low impact when loneliness was accounted for. Technology use was related to higher HRQoL, aligning our findings with the results of previous studies [9]. However, the magnitude of the positive effect was notable when considering prepandemic studies [10]. Loneliness impacted HRQoL even when social isolation and technology use were accounted for, in agreement with previous literature [10]. The cross-sectional design prevented us from determining causality and was the main limitation of this study. Our study has relevant implications for health professionals such as health psychologists seeking to improve the HRQoL of older adults, especially through adverse life events like the pandemic or other circumstances that would put older adults in a similar situation where their mobility has been restricted. Our study informs that loneliness should be addressed, in conjunction with increasing technology use, in interventions. The absence of longitudinal studies examining the same cohort before and after the pandemic makes this interpretation speculative. Further research is needed to determine causes, and future studies need to examine pandemic-linked long-term impacts on the mental health and well-being of older adults.

## Abbreviations

HRQoL: health-related quality of life.
